# Effect of Specific Spoilage Organisms on the Degradation of ATP-Related Compounds in Vacuum-Packed Refrigerated Large Yellow Croaker (*Larimichthys crocea*)

**DOI:** 10.3390/foods13131989

**Published:** 2024-06-24

**Authors:** Bohan Chen, Qi Yan, Tiansheng Xu, Dapeng Li, Jing Xie

**Affiliations:** 1 College of Food Science and Technology, Shanghai Ocean University, Shanghai 201306, China; cbobo0429@163.com (B.C.); qiyan000129@163.com (Q.Y.); xutiansheng44@163.com (T.X.); jxie@shou.edu.cn (J.X.); 2 Research Center of Aquatic-Product Processing & Preservation, Shanghai Ocean University, Shanghai 201306, China; 3 Key Laboratory of Aquatic Products High-Quality Utilization, Storage and Transportation (Co-Construction by Ministry and Province), Ministry of Agriculture and Rural Affairs, Shanghai Ocean University, Shanghai 201306, China

**Keywords:** seafood, quality deterioration, ATP-related compounds, spoilage microorganisms, enzyme activities

## Abstract

This study examined the spoilage potential of specific spoilage organisms on the degradation of adenosine triphosphate (ATP)-related compounds in vacuum-packed refrigerated large yellow croaker. The total viable count (TVC), ATP-related compounds and related enzymes of vacuum-packed refrigerated large yellow croaker inoculated with different bacteria (*Pseudomonas fluorescens* and *Shewanella putrefaciens*) were characterized using the spread plate method, high-performance liquid chromatography and assay kits, respectively. Results indicated that the TVC for both control and *Shewanella putrefaciens* groups reached spoilage levels at days 9 and 15, respectively. The changes of adenosine triphosphate (ATP), adenosine diphosphate (ADP), adenosine monophosphate (AMP) and adenosine deaminase activity across all groups showed no significant difference attributable to microbial growth. The results suggested that ATP to inosine monophosphate (IMP) degradation primarily occurs via fish’s endogenous enzymes, with minimal microbial involvement. On day 12, the IMP content in fillets inoculated with *Pseudomonas fluorescens* (0.93 μmol/g) was half higher than in those inoculated with *Shewanella putrefaciens* (0.57 μmol/g). Both spoilage organisms facilitated IMP degradation, with *Shewanella putrefaciens* making a more substantial contribution. Analysis of K values and correlation coefficients revealed that *Shewanella putrefaciens* was the primary factor in the freshness loss of refrigerated vacuum-packed large yellow croaker. These findings offer a reference for understanding quality changes in refrigerated large yellow croaker, especially regarding umami degradation at the microbial level.

## 1. Introduction

Large yellow croaker (*Larimichthys crocea*) is popular among consumers for its tasty and nutritious meat [1]. However, the richness of protein, carbohydrate and water in large yellow croaker creates excellent conditions for microorganisms to reproduce. Consequently, fresh large yellow croaker is susceptible to attack by specific spoilage organisms (SSO), resulting in spoilage, waste of resources and food safety problems [2]. Additionally, SSO attacks lead to fish flavor deterioration, notably freshness loss and bad odor development [3]. A large number of studies have shown that the shelf-life of large yellow croaker can be effectively extended by vacuum preservation [4]. However, some spoilage microorganisms can still grow even in vacuum packaging, which can damage the quality of the fish. Most studies have concentrated on inhibiting microbial growth to extend the shelf life of fish fillets. However, fewer studies have explored how microorganisms contribute to fish fillet spoilage. Thus, investigating the mechanism of microbial action in fish fillet spoilage is essential.

Microbial composition during fish storage varies with the storage environment, and SSOs differ among fish species, handing and storage conditions. It has been found that the specific spoilage organisms for refrigerated grass carp fillets include *Aeromonas* and *Pseudomonas* [5]; for large yellow croaker, *Pseudomonas* and *Shewanella* [6]; and for vacuum-packed sea bass, *Stenotrophomonas maltophilia* [7]. In a suitable environment, microorganisms grow and multiply, resulting in nutrient loss and deterioration in fish [5]. However, most of the current studies have focused on the activity of microorganisms in causing changes in fish proteins. Meanwhile, the impact of microorganisms on fish flavor degradation and the related degradation pathways and intermediate metabolites remain underexplored.

The degradation of adenosine triphosphate (ATP) and its associated products is an important biochemical change during fish storage by the following pathway: ATP → adenosine diphosphate (ADP) → adenosine monophosphate (AMP) → inosine monophosphate (IMP) → hypoxanthine ribonucleoside (HxR) → hypoxanthine (Hx). Studies have shown that the degradation product of ATP, IMP, is an important source of fish freshness. However, microbial utilization of IMP leads to spoilage substances like HxR, Hx, and uric acid (Ua), resulting in flavor loss. This in turn leads to the loss of freshness and bitterness of fish [8]. The degradation of ATP and its associated products is closely linked to microbial growth and reproduction, while different microorganisms affect the degradation pathways differently. However, the effects of various specific spoilage bacteria on the degradation of ATP and its associated products during cold storage of vacuum-packed large yellow croaker have not been clearly described.

In this study, we measured the changes in total viable count (TVC), ATP-related compounds content and related enzymes activity to investigate the ATP-related degradation pattern during storage of large yellow croaker inoculated with *Pseudomonas fluorescens* and *Shewanella putrefaciens*, as well as the microbial influence on the degradation of ATP-related compounds. This study’s findings offer theoretical guidance for processing, storing, and transporting aquatic products.

## 2. Materials and Methods

### 2.1. Preparation of Specific Spoilage Organisms

This study utilized two strains, *Pseudomonas fluorescens* and *Shewanella putrefaciens*, isolated from spoiled large yellow croaker in our previous work [9]. The strains were preserved in 30% glycerol and placed in a −80 °C refrigerator. Each strain was inoculated at 2% inoculum in 10 mL of TSB mixed in test tubes and incubated in a shaking incubator for 9 h (30 °C, 200 rpm). The total number of viable bacteria was measured every hour. Based on the results, the bacterial concentration reached approximately 9.0 log CFU/mL after 9 h. The incubated culture solution was diluted to 6.0 log CFU/mL using saline and used for subsequent inoculation experiments.

### 2.2. Sample Processing

Large yellow croaker (weight 0.61 ± 0.16 kg; length 34.2 ± 3.5 cm) were purchased from the Luchao Gang seafood markets (Shanghai, China) and then transported to the laboratory alive in an oxygenated foam box filled with water. The fish were stunned by striking their heads. The viscera and skin of the large yellow croaker were removed, and the fillets were taken from the front and back sides, washed and cut into four similarly sized fillets (40 ± 6 g). All procedures were performed in the instructions of the World Organization for Animal Health (OIE) guidelines. The fillets were washed three times with cold sterile water and wiped with sterile paper immediately.

### 2.3. Preparation of Sterile Fish Fillets

The fish fillets were immersed in a 5% (*w*/*v*) formalin solution for 40 s, followed by sterilization through triple rinsing with sterile water. Then fillets were dried and exposed to an ultraviolet lamp for 20 min. The sterilized fish fillets were subsequently stored at 4 °C until bacterial inoculation.

### 2.4. Bacterial Inoculation

Four groups of vacuum-packed fish fillets treated in different ways were set up in this study. The strains (*Pseudomonas fluorescens* and *Shewanella putrefaciens*) were inoculated into tryptic soy broth (TSB) and cultured to 8.0 log CFU/mL, respectively. Subsequently, the bacterial suspension was diluted to 6.0 log CFU/mL with 0.9% saline, and the sterile fillets were immersed in it for 10 min. The fillets were then washed with sterile water three times to achieve an initial total viable count level of about 4.0 log CFU/g and clean off disturbances on the fillets. For the control group (CK), unsterilized fish fillets were used, which were immersed in a sterile 0.9% sodium chloride solution for 10 min. In addition, fillets in the Sterility group were sterile and immersed in a sterile 0.9% sodium chloride solution for 10 min. All samples were vacuum packaged (MAP-JY500, Shanghai JIYI Machinery Co., Ltd., Shanghai, China) with high density polyethylene bags and stored in a refrigerator at a temperature of 4 ± 1 °C. For analysis, three fillets were randomly selected from each group and tested on days 0, 3, 6, 9, 12 and 15.

### 2.5. Total Viable Count (TVC)

A plate count method was used to determine the microbial counts of large yellow croaker fish during refrigeration [10]. A 5 g fish sample was weighed, 45 mL of sterile saline was added, homogenized for 5 min and then 1 mL of sample homogenate was prepared in 10-fold serial dilution with sterile saline. Two suitable dilutions of sample homogenate were selected and 100 μL of homogenate was drawn onto a plate-counting agar plate and spread evenly. The plates were incubated in an incubator at 30 °C for 48 h and then counted. Microbial counts are expressed as log CFU/mL with a minimum limit of 2.0 log CFU/mL.

### 2.6. Analysis of ATP-Related Compounds

Following the method of Li et al. [11] with minor modifications, 5.0 g of the sample was accurately weighed and homogenized (T 25 D S25, IKA, Staufen, Germany) by adding 10 mL of 10% perchloric acid (PCA). The mixture was then centrifuged (H1850R, Hunan Xiang Yi Laboratory Instrument Development Co., Ltd., Xiangtan, China) at 4 °C for 10 min at 8000 r/min. This procedure was repeated for the precipitate, and the supernatants were combined. Subsequently, the pH of the supernatants was adjusted to 6.5 using 10 mol/L KOH solution and fixed to 50 mL. The supernatant was filtered with 0.22 μm membrane. The filtrate was stored in a liquid-phase injection vial at −80 °C in a freezer for measurement.

The sample was measured by high-performance liquid chromatography (Waters E2695, Co., Milford, CT, USA) on a VP-CDSC18 (4.6 mm × 250 mm) column with 0.05 mol/L phosphate buffer at pH 6.0. The sample volume was 10 μL, the flow rate was 1 mL/min, the column temperature was 30 °C, and the detection wavelength was 254 nm. The standard curve was performed by determining the corresponding peak areas using Empower 3 software (Waters, Co., Milford, CT, USA) of different gradient concentrations of standard samples (ATP, ADP, AMP, IMP, HxR, and Hx). The ATP-related compounds content in samples was calculated from the peak area by Microsoft Office Excel 2023 based on standard curve and expressed in μmol/g.

### 2.7. Determination of Adenosine Deaminase (AMPD), Acid Phosphatase (ACP), Alkaline Phosphatase (ALP), 5′-Nucleotidase (5′-NT) and Xanthine Oxidase (XOD) Activity

An amount of 1.0 g of fish samples was accurately weighed, added to 9 mL of 0.9% saline and homogenized for 30 s using a homogenizer to make a 10% tissue homogenate. The protein content of a portion of homogenate was determined as described by Jiang et al. [12]. Briefly, 1 mL of protein solution was added to 4 mL of Biuret Protein Reagent and the reaction was carried out at 25 °C for 20 min. The absorbance was determined at 540 nm. The homogenate was centrifuged at 3000 r/min for 10 min. The supernatant was collected and used to determine enzyme activities. The activities of ACP, ALP and XOD were determined using the ACP, ALP and XOD assay kits (Nanjing Jiancheng Institute of Biological Engineering, Nanjing, China) according to the method described by Li et al. [13] and Li et al. [14]. 5′-NT activity was determined using the 5′-NT assay kit (Beijing Solarbio Technology Co., Ltd., Beijing, China) and AMPD activity was determined using the AMPD assay kit (Shanghai KEAIB Biotechnology Co., Ltd., Shanghai, China). Enzyme activity was determined according to the assay kit method and expressed in U/gprot.

### 2.8. Calculation of K-Value and Other Relevant Values

After obtaining the content of ATP-related compounds by HPLC, the K-value and other relevant values were calculated by the following equations [15]:

(1) $$K-value\;\left(\%\right)=\frac{HxR+Hx}{ATP+ADP+AMP+IMP+HxR+Hx}\times 100$$

(2) $$Ki-value\;(\%)=\frac{HxR+Hx}{IMP+HxR+Hx}\times 100$$

(3) $$H-value\;(\%)=\frac{Hx}{AMP+IMP+HxR+Hx}\times 100$$

(4) $$P-value\;(\%)=\frac{HxR+Hx}{AMP+IMP+HxR+Hx}\times 100$$

(5) $$Fr-value\;(\%)=\frac{IMP}{IMP+HxR+Hx}\times 100$$

(6) $$G-value\;(\%)=\frac{HxR+Hx}{AMP+IMP+HxR}\times 100$$

### 2.9. Statistical Analysis

All experiments were performed in triplicate and the experimental data were analyzed for significance using IBM SPSS Statistics 25 software (SPSS Inc., Chicago, IL, USA), and the experimental values were expressed as mean ± standard deviation (mean ± SD) and plotted using Origin 8.5 software (Origin Lab Co., Northampton, MA, USA). All data were subjected to one way ANOVA followed by the Duncan method with a significance level of 5%.

## 3. Results

### 3.1. TVC

**Figure 1:**
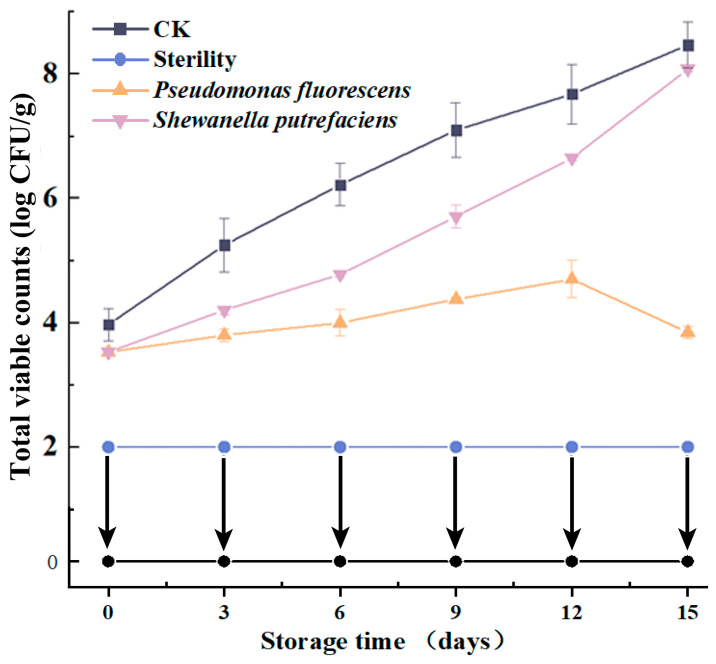
Changes in the total viable counts in vacuum-packed large yellow croaker fillets inoculated with different spoilage bacteria during refrigeration. (CK: unsterilized fish fillets; Sterility: sterilized fish fillets; *Pseudomonas fluorescens*: sterilized fish fillets inoculated with *Pseudomonas fluorescens; Shewanella putrefaciens*: sterilized fish fillets inoculated with *Shewanella putrefaciens*).

The variation in TVC of vacuum-packed large yellow croaker fillets is shown in Figure 1. Bacteria are a major cause of fish spoilage. Fish are considered spoiled and inedible above 7.00 log CFU/g [16]. The initial TVC of the CK group (3.95 ± 0.24 log CFU/g) indicates that the large yellow croaker used in this experiment was of a very fresh quality. The initially present bacteria primarily in the CK group were identified as genus *Shewanella*, *Psychrobacter*, *Lactococcus* and *Pseudomonas* and showed a low level [9]. Throughout the storage period, the large yellow croaker fillets of the Sterility group remained below the detection limit (2.00 log CFU/g), considering that bacteria have a negligible effect on their quality [3]. Meanwhile, the inoculation amount of large yellow croaker fillets in the inoculated group was about 3.50 log CFU/g at the initial stage, which was not significantly different from that of the CK group, indicating that the inoculation was successful. The inoculation amount demonstrated the tendency for microbial growth on fillets and highlighted the relationship between microbial growth and fillet quality. The bacteria are initially present primarily on the surface of the fillets and gradually enter the interior of the fillets as the storage time increases in the inoculated fillets. The TVC in the CK group rose quickly, hitting 7.08 ± 0.44 log CFU/g by day 9 (indicating spoilage) and 8.41 ± 0.37 log CFU/g by day 15 (indicating severe spoilage). As shown in Figure 1, sterile large yellow croaker inoculated with *Pseudomonas fluorescens* showed slower microbial growth, while microbial growth of sterile large yellow croaker fillets inoculated with *Shewanella putrefaciens* was faster. Throughout the storage process, the TVC of *Pseudomonas* did not exceed 5.00 log CFU/g. Meanwhile, a previous study had realized that *Shewanella* dominated the spoilage in vacuum-packed large yellow croaker fillets [9].

### 3.2. Changes in ATP-Related Components

**Figure 2:**
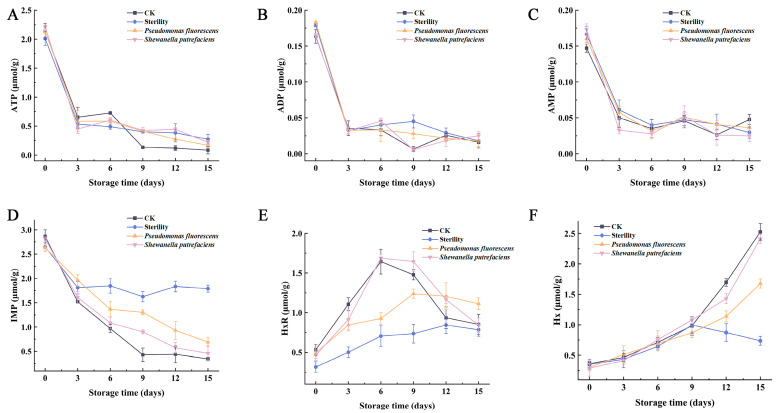
Changes in ATP (**A**), ADP (**B**), AMP (**C**), IMP (**D**), HxR (**E**) and Hx (**F**) in vacuum-packed large yellow croaker fillets inoculated with different spoilage bacteria during refrigeration. (CK: unsterilized fish fillets; Sterility: sterilized fish fillets; *Pseudomonas fluorescens*: sterilized fish fillets inoculated with *Pseudomonas fluorescens*; *Shewanella putrefaciens*: sterilized fish fillets inoculated with *Shewanella putrefaciens*).

In living fish, ATP continuously interconverts with ADP in cells, maintaining a dynamic equilibrium [17]. Following fish death, ATP loses its stable energy source as intracellular oxygen depletes and glycogen undergoes anaerobic fermentation to lactic acid, leading to catabolism by endogenous enzymes and the production of secondary metabolites. The degradation of ATP to IMP during fish storage typically occurs within 1 day, a finding corroborated by this study of Chen et al. [8]. As shown in Figure 2A–C, the contents of ATP, ADP and AMP decreased rapidly during the first day of storage. And the contents of ADP and AMP were lower throughout the storage period because of their rapid degradation as intermediate products of ATP degradation. Meanwhile, the contents of ATP, ADP and AMP were similar in all groups during the pre-storage period. This result suggests that microorganisms have less influence on the degradation process of ATP to IMP in large yellow croaker. It has been shown that several enzymes are involved in the process of ATP degradation to AMP, but these enzymes are from the fish itself and are not related to microorganisms [15].

IMP degradation was slower across all groups during the initial 3 days of storage, without significant differences, suggesting it was predominantly driven by endogenous enzymes. According to Figure 2D, obviously, microbial growth and reproduction were in the early stage, and the degradation of IMP was weak by microorganisms. After 3 days of storage, IMP began to decline in all groups, and the most obvious decreasing trend was in the CK group, with the content of only 0.35 ± 0.04 μmol/g on the 15th day. The decline in IMP mirrored the rise in TVC. In the Sterility group, IMP content reduced to 1.79 ± 0.07 μmol/g, indicating that endogenous enzymes degraded IMP less than microorganisms did. The differences exhibited in the CK and Sterility groups at the later stages of storage coincided with the time of microbial proliferation, suggesting that specific spoilage microorganisms were the main cause of IMP degradation. *Shewanella putrefaciens* promoted IMP degradation, and the rate of IMP degradation in group *Shewanella putrefaciens* was second only to that of group CK. This suggested that *Shewanella putrefaciens* played a major role in vacuum packaging, maybe due to the fact that *Shewanella putrefaciens* can still proliferate in vacuum packaging. Conversely, *Pseudomonas fluorescens* also facilitated IMP degradation, albeit at a slower rate, aligning with its TVC in vacuum packaging. It indicates that the biomass of spoilage microorganisms is also an important factor influencing the degradation of IMP, and specific spoilage microorganisms can proliferate in a suitable environment, thus accelerating the degradation of IMP [18].

The degradation of IMP continues during the middle and late stages of fish storage to produce HxR and Hx. Moreover, it is generally accepted that HxR and its degradation products (Hx, Xa and Ua) are spoilage substances, and their presence severely reduces the quality and flavor of fish products [19]. Accumulated Hx, along with certain amino acids and peptides, has been linked to meat’s bitter flavor [20]. According to Figure 2E, the initial value of HxR in the CK group was 0.53 ± 0.07 μmol/g during storage, and the HxR content first increased and then decreased to 0.85 ± 0.13 μmol/g on day 15. The *Shewanella putrefaciens* group showed a similar trend to the CK group, but the decrease was more blocked in the CK group, which might be due to the fact that the large yellow croaker fillet inoculated group in the same time period had fewer colonies and lower enzyme-producing activity than the CK group. The HxR content in the Sterility group increased gradually over the first 6 days of storage to 0.70 ± 0.13 μmol/g and was stabilized. The content of HxR in group *Pseudomonas fluorescens* showed a similar trend to that in group Sterility. The results indicate that specific spoilage bacteria are linked to HxR degradation, with *Shewanella putrefaciens* degrading HxR more significantly than *Pseudomonas fluorescens*. The present study also demonstrated that the enzymes contributing to the degradation of HxR were mainly from microorganisms. It explained the positive correlation between the number of microorganisms and the rate of degradation of IMP in each group at the late stage of storage, resulting in a greater rate of HxR production as well as accumulation content in the CK and *Shewanella putrefaciens* groups than in the Sterility and *Pseudomonas fluorescens* groups. During late storage, microbial action led to exogenous enzymes augmenting endogenous ones, increasing HxR degradation in the CK and *Shewanella putrefaciens* groups beyond production rates and thus reducing HxR levels gradually [21]. In contrast, the degradation of HxR in the Sterility group was mainly by endogenous enzymes, and the rate of production was greater than the rate of degradation, resulting in a period of HxR accumulation during this period. Due to *Pseudomonas fluorescens*’s slower HxR degradation rate, the *Pseudomonas fluorescens* group experienced higher HxR accumulation compared to the CK and *Shewanella putrefaciens* groups in the end. Figure 2F shows that the initial content of Hx in the CK group was 0.36 ± 0.03 μmol/g, rising throughout storage to 2.52 ± 0.14 μmol/g by day 15. In the Sterility group, HxR slowly increased for the first 9 days of storage, similar to the CK group, with levels fluctuating between 0.73–0.99 μmol/g afterwards, indicating a slow decomposition of HxR and a minor role for endogenous enzymes. This demonstrates that Hx accumulation is primarily linked to microorganisms, as evidenced by the *Pseudomonas fluorescens* and *Shewanella putrefaciens* groups. Liu et al. [22] proved that *Shewanella* and *Pseudomonas* secrete nucleoside phosphorylase, and Hernández-Cázares et al. [23] have reported that the formation of Hx is related to nucleoside phosphorylase produced by spoilage bacteria. In late-stage fish spoilage, Hx decomposes into uric acid; however, this study found minimal uric acid content, suggesting no significant accumulation during storage, with potential for uric acid production after prolonged periods.

### 3.3. Changes in the Viability of Enzymes Involved in ATP Degradation 

**Figure 3:**
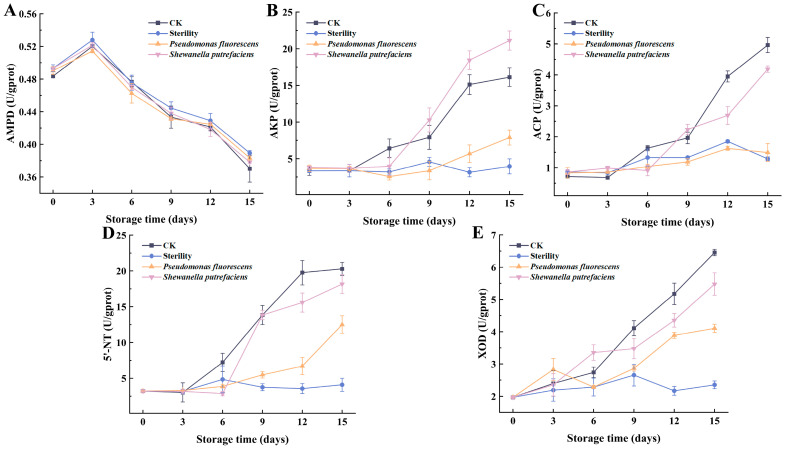
Changes in AMPD (**A**), AKP (**B**), ACP (**C**), 5′-NT (**D**) and XOD (**E**) in vacuum-packed large yellow croaker fillets inoculated with different spoilage bacteria during refrigeration. (CK: unsterilized fish fillets; Sterility: sterilized fish fillets; *Pseudomonas fluorescens*: sterilized fish fillets inoculated with *Pseudomonas fluorescens*; *Shewanella putrefaciens*: sterilized fish fillets inoculated with *Shewanella putrefaciens*).

Adenosine deaminase (AMPD) is an important catalyzing enzyme for IMP accumulation, mainly controlling the formation of IMP during the degradation of ATP, and studies have shown that the main function of AMPD is to produce IMP and NH_3_ by catalyzing the deamination of AMP [24]. As shown in Figure 3A, there was no significant difference in AMPD activity among the groups, which reached the highest value on day 3 and then gradually decreased to the lowest on day 15. This aligns with findings of Li, Zhang, Song, Wang, Kong and Luo [15], where AMDP enzyme activity increased during the pre-storage period before beginning to decline, with no significant difference in AMDP viability across groups. The results indicated that the degradation of AMP and the generation of IMP are mainly the action of endogenous enzyme AMPD, and the specific spoilage bacteria have less influence on this biochemical process.

AKP, ACP and 5’-NT enzymes are the primary phosphohydrolases involved in IMP degradation [8]. As shown in Figure 3B, after 3 days of storage, there was a significant difference in AKP activity among the four groups, and AKP activity was significantly higher in the CK and *Shewanella putrefaciens* groups than in the Sterility and *Pseudomonas fluorescens* groups at the later stages of storage. The results indicated that AKP was produced through microbial secretion in addition to originating from the fish itself, and *Shewanella putrefaciens* produced higher AKP activity than *Pseudomonas fluorescens* under vacuum. Figure 3C shows no significant differences in ACP activity among groups in the initial 3 days of storage, indicating minimal microbial impact on ACP activity. This is likely due to fewer microbes present, with ACP predominantly originating from the fish itself. After 6 days of storage, the significant difference in ACP activity was higher in the CK and *Shewanella putrefaciens* groups than in the other groups. The highest ACP activity values in the late storage period were less than 5.00 U/gprot, well below the AKP activity values. This indicates that AKP has a stronger ability to degrade IMP degradation than ACP in large yellow croaker. As shown in Figure 3D, the initial value of 5′-NT activity in fresh large yellow croaker was 3.22 ± 0.17 U/gprot. The results showed no significant difference in the change of 5′-NT activity in each group during the first 3 days of storage. In the late storage stage, the 5′-NT activity of each group was significantly higher than that of Sterility group. The 5′-NT activity of the CK group showed a rapidly increasing trend after 3 days and reached 20.28 ± 0.93 U/gprot in the end. 5′-NT originated from the fish itself; in the middle and late stages spoilage bacteria also secreted 5′-NT and spoilage bacteria and gradually dominated. The 5′-NT activity in the Sterility group was kept at a low level, and showed no significance through the storage of large yellow croaker fillets. Considering the changes in IMP content and ACP and AKP activities, IMP levels in the Sterility group remained nearly constant in the late storage stage, whereas they declined in other groups, indicating that spoilage bacterial enzymes primarily drive IMP degradation [17].

XOD can catalyze the hydrolysis of Hx to generate uric acid. Figure 3E shows the changes of XOD activity during 4 °C refrigeration of vacuum-packed large yellow croaker. Initially, there was no significant difference in XOD activity between the CK and Sterility groups, but from days 6–15, the CK group’s activity was significantly higher, suggesting a microbial influence on XOD activity. Within 3 days, there was no significant change in XOD activity in both groups because the microbial influence on the fish meat was small at this time. With the extension of storage time, the XOD activity increased in the CK group, while it fluctuated in the range of 1.97–2.65 U/gprot in the Sterility group. This suggests microorganisms facilitate XOD production, with Figure 3E showing *Shewanella putrefaciens* possessing greater XOD production capacity than *Pseudomonas fluorescens*, highlighting its role as a primary enzyme activity source [21]. In this study, stored large yellow croaker produced very little uric acid, which may be due to the low amount of Hx hydrolyzed, or Hx mainly produced xanthine and had not yet produced a large amount of uric acid.

### 3.4. K Value and Other Relevant Values

**Figure 4:**
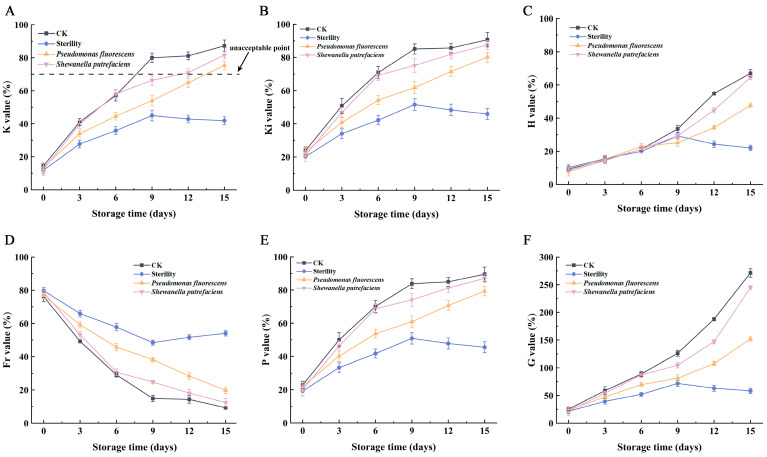
Changes in K (**A**), Ki (**B**), H (**C**), Fr (**D**), P (**E**) and G (**F**) values in vacuum-packed large yellow croaker fillets inoculated with different spoilage bacteria during refrigeration. (CK: unsterilized fish fillets; Sterility: sterilized fish fillets; *Pseudomonas fluorescens*: sterilized fish fillets inoculated with *Pseudomonas fluorescens*; *Shewanella putrefaciens*: sterilized fish fillets inoculated with *Shewanella putrefaciens*).

The K value, the ratio of HxR and Hx to the total of six ATP degradation products, is used to assess fish freshness. Fresh fish have K values below 20%, moderately fresh fish range from 20% to 50%, and fish with K values over 70% are considered inedible [25]. The changes in K values for large yellow croaker are shown in Figure 4A. Initial K values were around 16%, suggesting the fish was extremely fresh. As storage time extended, K values increased. Similarly to the previous results, the K values of the CK and *Shewanella putrefaciens* groups increased faster, exceeding 50% on the sixth day simultaneously, indicating that the fish was no longer fresh at this point, and the K value exceeded 70% on the ninth day, making the fish inedible. However, the K values of the Sterility group increased more slowly, and at the end of storage, the K value of the fish was lower than 50%, indicating that microbial growth was the main factor in the loss of fish freshness [26,27]. The K value growth rate of group *Pseudomonas fluorescens* was lower than that of groups CK and *Shewanella putrefaciens*, due to the following factors: 1. *Pseudomonas fluorescens*’s struggle to survive and reproduce in vacuum conditions, impacting ATP-related products less than *Shewanella putrefaciens*; 2. the fact that *Pseudomonas fluorescens* degrades ATP-related products less effectively than *Shewanella putrefaciens*, likely due to lower enzyme activity. In this study, spoilage microorganisms mainly affected the conversion of IMP to HxR and Hx. The percentage of HxR and Hx in the total ATP-related products of K values can indicate the difference between the inoculated group and the control group. Given the similarities between the CK and *Shewanella putrefaciens* groups, *Shewanella putrefaciens*’s activity in vacuum packaging appears to be a key factor in fish spoilage. The Ki values differed from the K values [28]; however, in this study, ADP and AMP were degraded rapidly as ATP intermediary metabolites in small quantities, and therefore were similar to the K values.

In addition, there are H, P, Fr and G values that can also be used as indicators of fish freshness evaluation [29,30], calculated by Equations (3)–(6) [15]. H, P and G values increased with storage time while Fr values decreased with storage time. The Fr-value, representing the IMP percentage of the total IMP, HxR and Hx content, equates to 100% − Ki. The equation implies that higher IMP content results in more flavorful fish [31], thus Fr decreases as time extends. Both P and K values show the trend of the total level of HxR and Hx, so they are very similar, and the difference between H and G values and these three values is shown in the difference in Hx, with the H value indicating the percentage of Hx in the total content of IMP, HxR, and Hx, and the G value indicating the percentage of HxR and Hx in the total content of AMP, IMP and HxR. This study identifies K, Ki, H, P, Fr and G values as suitable freshness indicators for CK group fish samples, whereas only the K value is appropriate for assessing freshness in fish inoculated with spoilage bacteria.

## 4. Conclusions

This study evaluated the spoilage potential of *Shewanella putrefaciens* and *Pseudomonas fluorescens* in the degradation of ATP-related compounds and the quality changes of vacuum-packaged refrigerated large yellow croaker fillets. Microorganisms were identified as the minimal responsible for the degradation process of ATP to IMP, while they exhibited strong ability in IMP and its degradation products. *Shewanella putrefaciens* was identified as the primary bacteria responsible for the IMP degradation and increase in K value of large yellow croaker fillets. Meanwhile, the K value is deemed more suitable for assessing the freshness and flavor of vacuum-packaged refrigerated large yellow croaker in this study. This research highlighted the significance of inhibiting microbial growth to preserve the degradation of ATP-related compounds in vacuum-packaged refrigerated large yellow croaker fillets, especially in converting IMP to Hx. Furthermore, it provided theoretical guidance for aquatic product quality control and flavor management.

## Data Availability

The original contributions presented in the study are included in the article; further inquiries can be directed to the corresponding author.
